# Meeting of the Central New York Dental Association

**Published:** 1864-01

**Authors:** S. B. Palmer


### Proceedings of Societies.

### MEETING OF THE CENTRAL NEW YORK DENTAL ASSOCIATION.

This society held its first semi-annual meeting in Syracuse, on Tuesday, November 10th, 1863.

The President, Dr. S. Ball, in the Chair.

The minutes of the previous meeting, as read by the Secretary, were adopted.

An Essay—the subject having been given at the previous meeting—on the Importance of Preserving Natural Teeth, was read by Dr. S. B. Palmer.

A vote of thanks was given for the same; also, a request that copies be furnished the journals, for publication.

Dr. S. G. Martin, who was previously appointed to lead in discussion on the subject of the Essay, read a well-written paper embracing the various materials used in filling teeth; also, giving his method of using gold for that purpose.

Dr. F. O. Hyatt gave his experience and manner of using annealed gold-foil,—showing the result of his labors, by exhibiting fillings in the mouth of a patient present, where the crowns of the incisors which were badly broken away, had been fully restored to the original form by gold-fillings.

A vote of thanks was given to Drs. Martin and Hyatt—to the former for the paper written by him, and to the latter for the pains taken to satisfy the Association of the practicability of restoring teeth for usefulness, such as are too often doomed to the forceps.

The discussion became general and interesting,—showing that dentists have higher aims than merely to extract teeth, that they may supply their places artificially.

### EVENING SESSION.

President in the Chair. Subject of filling teeth resumed, including fang-filling and the treatment of exposed nerves.

Although various opinions were expressed as to the practicability of the latter treatment, the discussion plainly showed a wonderful advance in the preservation of teeth for usefulness, which, by many, are deemed worthless.

The discussion closed, at a late hour.

A committee was appointed to arrange the order of business for the next meeting; also, one to attend to other business of the Association.

The place of holding the annual meeting was fixed at Auburn, on the second Tuesday in May, 1864.

Association adjourned. S. B. PALMER. *Sec'y.*

